# Solid State Polymerization of Biodegradable Poly(butylene sebacate-co-terephthalate): A Rapid, Facile Method for Property Enhancement

**DOI:** 10.3390/polym15051133

**Published:** 2023-02-24

**Authors:** Daegyu Lim, Su-il Park

**Affiliations:** Department of Packaging, Yonsei University, Wonju 26493, Republic of Korea

**Keywords:** solid state polymerization, bio-based polymer, PBSeT, rheological propeties, rapid process

## Abstract

Poly(butylene sebacate-co-terephthalate) (PBSeT) has generated attention as a promising biopolymer for preparing bioplastics. However, there are limited studies on the synthesis of PBSeT, impeding its commercialization. Herein, with a view to addressing this challenge, biodegradable PBSeT was modified using solid state polymerization (SSP) with various ranges of time and temperature. The SSP used three different temperatures below the melting temperature of PBSeT. The polymerization degree of SSP was investigated using Fourier-transform infrared spectroscopy. The changes in the rheological properties of PBSeT after SSP were investigated using a rheometer and an Ubbelodhe viscometer. Differential scanning calorimetry and X-ray diffraction showed that the crystallinity of PBSeT was higher after SSP. The investigation revealed that after SSP for 40 min at 90 °C, PBSeT exhibited higher intrinsic viscosity (increased from 0.47 to 0.53 dL/g), crystallinity, and complex viscosity than PBSeT polymerized at other temperatures. However, a high SSP processing time resulted in a decrease in these values. In this experiment, SSP was most effectively performed in the temperature range closest to the melting temperature of PBSeT. This indicates that SSP could be a facile and rapid method for improving the crystallinity and thermal stability of synthesized PBSeT.

## 1. Introduction

In recent years, the importance and necessity of utilizing bioplastics has been increasing owing to the importance of carbon neutrality and concern for environmental pollution [1]. In particular, the possibility of resource drain and the need to replace petroleum-based plastics have also increased. These phenomena occur in the global plastics market, and the demand for bioplastics has also increased. According to the global plastics market, the market share of biopolymers is growing every year, and various types of bioplastics are currently being studied and synthesized. Polylactic acid (PLA) is an early biopolymer, and many related studies have been conducted [1,2,3]. In addition to PLA, biopolymers such as polyhydroxyalkanoate (PHA) [4,5,6], poly(butylene adipate-co-terephthalate) (PBAT) [7,8], and polybutyl succinate (PBS) are being studied [9,10]. Among them, PBAT is a typical biopolymer with properties of biodegradability and flexibility, and it has been commercially used in mulching films and compost bags [11,12]. However, adipic acid, the main material in PBAT, is a petroleum-based raw material that may affect environmental pollution [13,14]. Poly(butylene sebacate-co-terephthalate) (PBSeT), a promising polymer with high biodegradability [15] and ductility, is made from biomass-based sebacic acid, and it could replace the petroleum-based material in PBAT [16].

In the synthesis of bio-based polymers such as PBSeT and PBAT, the catalytic activity of metal–organic compounds can help accelerate the reaction of monomers. The main difference between the catalytic properties of metal–organic compounds and organic compounds is the presence of metal in the former [17]. Metal ions can act as Lewis bases, increasing their catalytic activity, whereas organic compounds lack this ability [18]. Furthermore, metal–organic compounds can also form strong bonds with other molecules and act as a bridge between two reactants, allowing them to interact more easily and quickly [19]. Ultimately, this leads to an increase in catalytic activity in comparison to that of organic compounds.

Several studies have reported the synthesis and properties of PBSeT. Jaisankar et al. [20] synthesized PBSeT using a direct melt polycondensation method with terephthalic acid (TPA) and studied its enzymatic degradability. Kim et al. [21] synthesized PBSeT using various ratios of sebacic acid and dimethyl terephthalate (DMT) by replacing TPA. The PBSeT synthesized in their study had a high molecular weight (Mw, 88,700–154,900 g/mol) and a good elongation at break with values greater than 1500%. Li et al. [22] reported the synthesis of PBSeT copolyesters using glycerol as a cross-linking agent and found that glycerol could improve the properties of PBSeT. While studying the synthesis and optimization of PBSeT properties, Kim et al. [21] also studied different methods to improve the properties of PBSeT by blending it with PLA, which showed reinforced mechanical strengths. Kwon et al. [23] blended PBSeT and PLA using poly(ethylene oxide) (PEO) as a compatibilizer. Interestingly, Jang et al. [24] successfully blended PBSeT with PLA by applying maleic anhydride-grafted PLA as a compatibilizer, which led to increased miscibility of PLA/PBSeT blends.

However, PBSeT is in the experimental stage, meaning that it is not in stable and constant supply. These drawbacks of PBSeT could be overcome through solid state polymerization (SSP) but research of applying SSP to PBSeT copolyesters has not been studied yet. In comparison with the conventional polymerization process, SSP is different in a number of ways. Conventional polymerization is concerned about excessive consumption of energy and time when molecular weight is increased above a certain level [25]. On the other hand, SSP progresses between the glass transition temperature (T_g_) and melting temperature (T_m_) of the polymer, which is in a temperature range lower than that of conventional polymerization and could improve the rheological characteristics and crystallinity of the polymer. Moreover, because no additional catalysts are needed, there is much less environmental impact [26].

The key parameters affecting the efficiency of SSP are temperature, time, and gas composition, which are mostly adjusted for extracting by-products and for the efficiency of SSP [26]. SSP is divided into direct SSP and post SSP. Direct SSP is a technique used in the synthesis of polymers such as nylon 6-6, etc. It is a polymerization method that maintains a solid state throughout the entire process [27]. Post SSP is used to increase the molecular weight of pre-synthesized prepolymers through additional processing. In industry, it has been used to increase the molecular weight and crystallinity of PET [28,29], and in the field of bioplastics, research on SSP for PLA is being actively conducted [30,31]. The SSP process can improve the properties of prepolymers and support environmental sustainability by reducing energy consumption [32]. In this research, PBSeT was synthesized and modified using SSP with various times and temperatures. We examined the practicality of SSP of relatively short duration by measuring the rheological properties and crystallinity of PBSeT.

## 2. Materials and Methods

### 2.1. Materials

DMT (>99.5%) was supplied from SK Chemical (Seoul, Republic of Korea). 1,4-Butanediol (BDO) (>99.5%) and extra pure grade sebacic acid were provided by Daejung Chemical & Metal Co., Ltd. (Siheung, Republic of Korea). Titanium tetrabutoxide (TBT) (97%) was purchased from Merck Co. (Darmstadt, Germany), and chloroform (>99.5%) was purchased from Honeywell (Charlotte, NC, USA).

### 2.2. Synthesis of PBSeT

**Figure 1:**
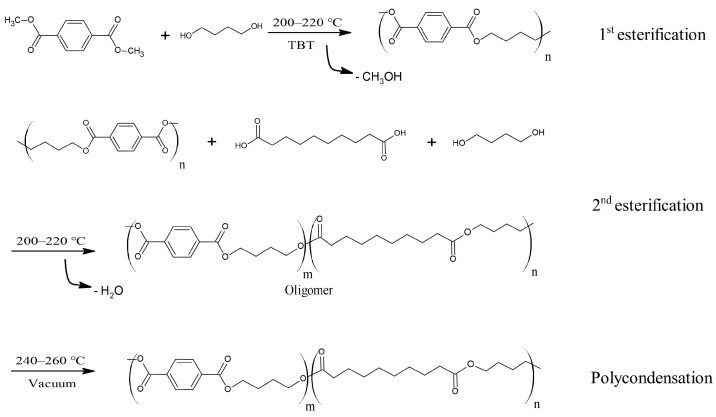
Schematic of PBSeT synthesis process.

In this study, PBSeT was successfully synthesized (T_m_: 93.4 °C) using DMT and sebacic acid [21]. TBT was used as a catalyst. The synthesis was performed in a 1 L reaction tank installed in a heating mantle. Esterification was conducted in two steps (Figure 1). In the first esterification step, 1.25 mol% of BDO, 0.4 mol% of DMT, and 0.37 g/mole of TBT were used. After the first step, 0.6 mol% of sebacic acid and 0.37 g/mole of TBT were added for second step. The temperature range for esterification was 200–220 °C, and the time range was 40–60 min, determined by the maximum temperature of the reaction tank. After the esterification process, the polycondensation step progressed at 240–260 °C, and the reactant was stirred strongly using a mechanical stirrer (MINISART 80, IKA, Germany). During the polycondensation step, the vacuum was gradually increased to maintain the vacuum pressure at less than 1 torr and the time period was 180 min. The total reaction time of this PBSeT synthesis process was shorter than other previous studies [22].

### 2.3. Solid State Polymerization (SSP)

**Figure 2:**
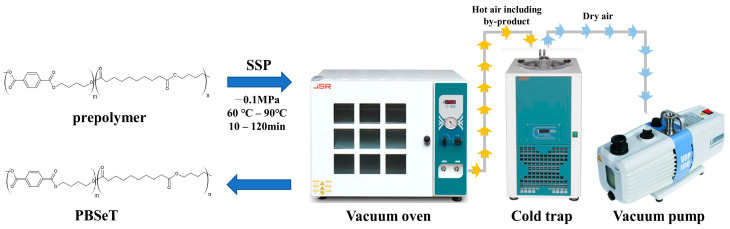
Process of SSP for PBSeT.

The SSP process was carried out in an oven-scale aluminum tray reactor with a width of 150 mm and a length of 185 mm at 60–90 °C with vacuum conditions maintained at 10 torr of pressure using a vacuum pump (VOP-100, Poongil commercial, Seoul, Republic of Korea). (Figure 2) The sample (5 g) was subjected to the SSP process, and the processing time was 10–120 min at each temperature; only one sample was processed at a time. All samples were sealed with silica gel and stored in a desiccator.

### 2.4. Characterization

Fourier-transform infrared (FT-IR) absorption spectra for the PBSeT polyesters were obtained using an FT-IR spectrometer (Spectrum65, PerkinElmer, Waltham, MA, USA) in ATR mode. FT-IR spectra were acquired after 32 scans and measured from 4000 to 400 cm^−1^. All samples were dissolved in chloroform and cast onto a glass plate. Casting of PBSeT was conducted in a fume hood, and all samples were dried at 23–25 °C for a day.

The intrinsic viscosity (*η*) of SSP processed samples was measured at 24–25 °C using a Ubbelohde viscometer (CuotaLab Sharing Korea, Seoul, Republic of Korea). All samples were dissolved in chloroform and filtrated with a single-use filter (Sartorius, Minisart, Germany). The intrinsic viscosity of each PBSeT sample was calculated according to the Billmeyer equation (Equation (1)).

(1) $$\left[\eta \right]=0.25\times \frac{{\left[2\{\frac{t}{t_{0}}-\ln\left(\frac{t}{t_{0}}\right)-1\}\right]}^{\frac{1}{2}}}{c}$$

where *c* is the concentration of polymer solution (g/dL), *t* is the flow time of the polymer solution (s), and *t*_0_ is the flow time of the pure solvent (s).

High-resolution X-ray diffraction (HR-XRD) spectra of the PBSeT samples were recorded using a high-resolution X-ray diffractometer, manufactured by Rigaku (Tokyo, Japan). The XRD measurement was carried out in the scattering range from 2θ = 0° to 2θ = 90°. Samples were prepared using a casting method with chloroform and were cut to 10 × 10 mm. The size of the PBSeT crystallites was calculated using the X-ray diffraction peaks and the Scherrer equation (Equation (2)) [33].

(2) $$\tau =\frac{K\gamma }{\beta \cos\theta }$$

where *τ* is the size of the crystallite, *β* is the line broadening at the full width at half maximum (FWHM) intensity, *K* is a dimensionless shape factor with a value close to unity, *γ* is the X-ray wavelength, and *θ* is Bragg’s angle.

To investigate the effect of SSP on thermal properties, differential scanning calorimetry (DSC) data were obtained from a DSC Q-20 apparatus (TA instruments, Milford, MA, USA). All samples weighed 5.5–5.6 mg and were sealed in an aluminum pan and lid. The samples were heated and cooled under a nitrogen flow in a temperature range of −50–180 °C at a heating rate of 20 °C/min. The crystallinity (*X_c_*) of the PBSeT was obtained using Equation (3).

(3) $$X_{c}\left(\%\right)=\left[\frac{\Delta H_{\mathrm{Se}}}{\Delta H_{\mathrm{Se}}^{100}}+\frac{\Delta H_{T}}{\Delta H_{T}^{100}}\right]\times 100$$

where $\Delta H_{Se}$ and $\Delta H_{T}$ are the melting enthalpies of the lower and higher melting temperatures of PBSeT, respectively [15], $\Delta H_{Se}^{100}$ is the estimated value for the 100% crystalline PBSe sample (210.8 J/g), and $\Delta H_{T}^{100}$ is the estimated value for the fully crystalline PBT sample (142 J/g) [15].

The thermal degradation properties of each PBSeT sample after the SSP process were analyzed by thermogravimetric analysis (TGA 4000, PerkinElmer, USA). Approximately 10 ± 1 mg of each specimen was heated from 30 °C to 800 °C at a rate of 15 °C/min under a nitrogen environment.

The shear viscosity was measured in a rotational shear rheometer (AR2000 EX, TA instruments, Milford, MA, USA) using a corn plate (diameter = 25 mm, gap = 1.1 mm). The strain amplitude was set to 1% for determining the linear viscoelastic region. The frequency range (ω) of the rheometer was set between 0.1 and 100 rad/s. The rheological characteristics of the PBSeT samples were measured in the molten state (120 °C). The shear complex viscosity (η*), storage modulus (G′) and loss modulus (G″) were measured. The samples were preheated at 120 °C for 3 min before the viscosity measurement.

## 3. Results

### 3.1. Effect of the SSP Process on PBSeT

**Figure 3:**
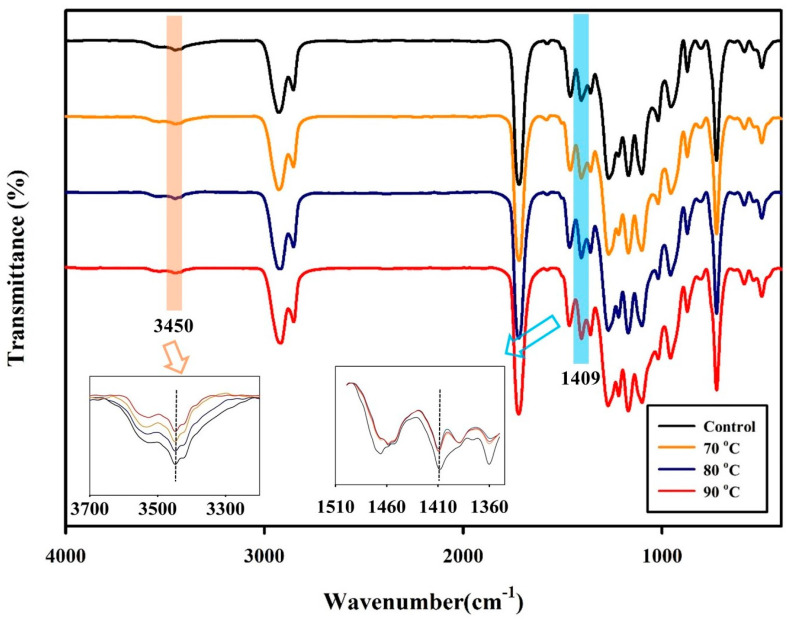
FT–IR spectra for PBSeT and PBSeT after SSP at 70, 80, and 90 °C for 40 min. (Insets: magnified 3450 and 1409 cm^−1^ bands).

As mentioned in Section 2.2, PBSeT was synthesized at lab scale with a two step polymerization using TBT as a catalyst. The FT-IR spectra shown in Figure 3 indicate that PBSeT was synthesized with a chemical structure similar to that of PBSeT reported previously [22,34]. The medium-intensity peaks at 2930 and 2850 cm^−1^ were attributed to C–H bonds. High-intensity peaks corresponding to C=O stretching were observed at 1714 cm^−1^, which is typical of aliphatic peaks. The peaks at approximately 3450 cm^−1^ indicate the O–H stretching of alcohol, and the O–H bending of carboxylic acid was observed at 1409 cm^−1^. According to the magnified FT-IR transmittance of the 3450 and 1409 cm^−1^ bands, the transmittance peak of the O–H groups of alcohol and carboxylic acid decreased after SSP. These may indicate that the degree of polymerization of PBSeT is increased through SSP. As SSP proceeds, various by-products and unreacted end groups disappear, and the concentration of O–H end groups of alcohol and carboxyl groups decrease accordingly [35].

### 3.2. Changes in Rheological Properties of PBSeT after the SSP Process

**Figure 4:**
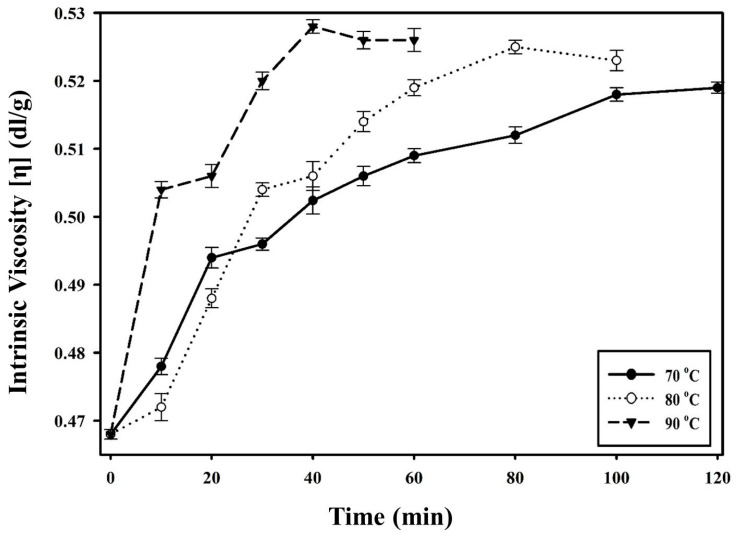
Change in intrinsic viscosity of PBSeT samples during the SSP process at different temperatures.

The changes in the rheological behavior of PBSeT after the SSP process can be verified by the intrinsic viscosity. As shown in Figure 4, intrinsic viscosity increased with increasing temperature of the SSP process. After the SSP process, the *η* values of the samples increased gradually at all temperature ranges after 40 min. The *η* of PBSeT increased as the SSP temperature increased. At 90 °C, a temperature close to the T_m_ of PBSeT, the *η* of PBSeT showed the highest initial ascent among all tested temperatures. SSP is typically conducted in a temperature range between the T_g_ and T_m_ of a polymer in solid state, and efficiency increases as the temperature approaches T_m_, which is a higher temperature [36,37]. The higher temperature further enhances the mobility of the macromolecular chains of PBSeT, which increases the reactivity of the hydroxyl and carboxyl end groups. This result demonstrates that SSP can be used to improve the rheological properties of PBSeT in an environmentally friendly manner over a relatively short duration without using an additional catalyst [38,39,40]. In the SSP procedure, the time for the *η* value to reach its maximum was 120, 80, and 40 min at 70, 80, and 90 °C, respectively. This was the result of the higher SSP temperature providing more energy, and it induced a higher SSP reaction rate. In addition, the higher SSP temperature led to more movement in the amorphous region of the polymer, which led to greater end-chain reaction. However, the *η* value of PBSeT after the SSP process at 80 and 90 °C slightly decreased after it reached its maximum value. It could be supposed that thermal degradation, which decreases the viscosity of the polymer, occurred in polymer chains from excessive energy and prolonged SSP time. Therefore, SSP at sufficiently high temperatures with appropriate duration can effectively elevate the *η* value of PBSeT.

**Figure 5:**
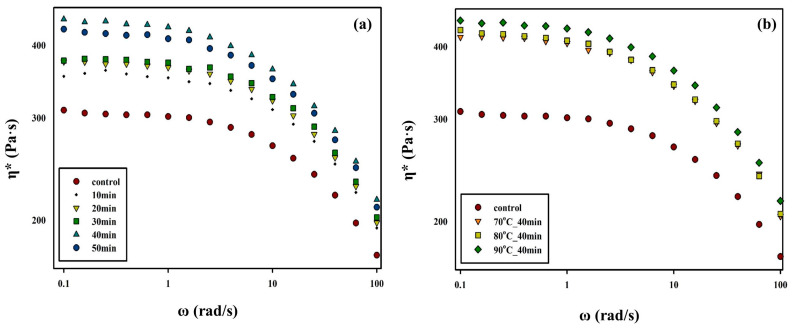
Complex viscosity (η*) of PBSeT at T = 120 °C: (**a**) complex viscosity of the PBSeT sample that underwent SSP at 90 °C for different lengths of time, (**b**) PBSeT sample that underwent SSP at different temperatures for 40 min.

Figure 5 indicates the changes in the complex viscosity, η*, of PBSeT after the SSP process using the rheometer data; the highest η* value in the short term and the effective improvement are verified at the processing times (at a constant temperature of 90 °C) and temperatures (for a constant processing time of 40 min) of the SSP process. The rheological behavior, such as the complex viscosity of a polymer, is an important factor that indicates polymer properties, such as the degree of polymerization [41]. The complex viscosity of PBSeT decreased as the frequency range increased, emulating the shear thinning behavior of typical thermoplastic polymer melts [22]. As the SSP processing time increased, the complex viscosity increased and then slightly decreased when the SSP was conducted at 90 °C for 50 min (Figure 5a).

**Figure 6:**
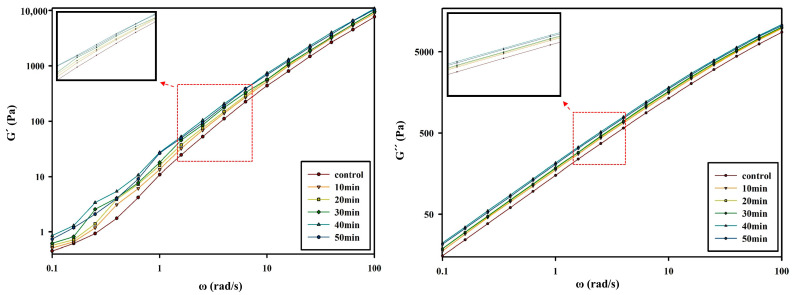
Storage modulus and loss modulus of PBSeT samples that were SSP processed at 90 °C for different periods of time. The data were acquired at T = 120 °C.

The storage modulus and loss modulus are fundamental analysis criteria that represent the viscoelastic characteristics of polymers. In addition, the storage modulus and loss modulus of PBSeT showed a bias similar to the complex viscosity of PBSeT. In Figure 6, the storage modulus and loss modulus of the PBSeT sample that underwent SSP at 90 °C at various times can be observed. They increased sharply for the samples subjected to SSP for 10, 20, 30, and 40 min but decreased slightly for the sample subjected to 50 min of SSP.

### 3.3. Melting and Crystallization Behaviors of PBSeT after SSP Process

**Figure 7:**
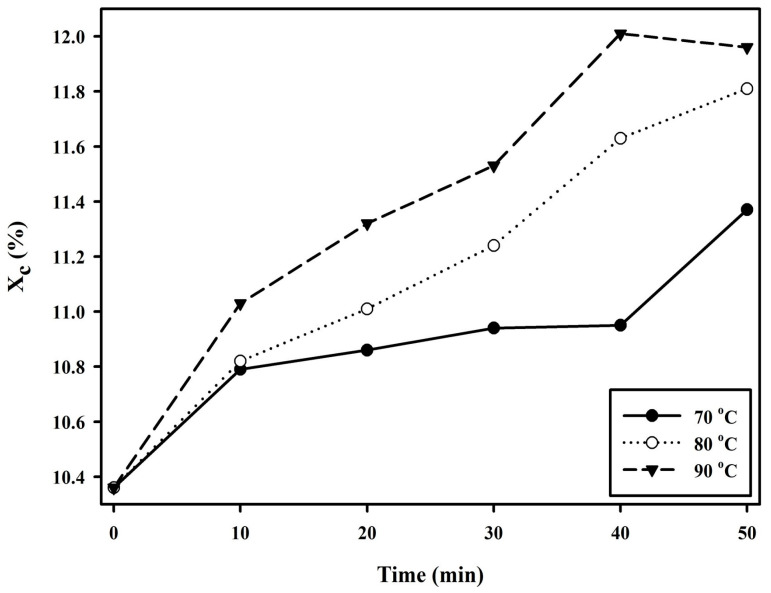
Evolution of the degree of crystallinity (X_c_) for different times and temperatures of SSP on PBSeT.

The thermal characteristics of PBSeT after the SSP process at different times and temperatures were determined by DSC and TGA, and the results are presented in Table 1. After the SSP procedure, the T_g_ of PBSeT was slightly reduced as the temperature of the SSP process increased owing to a reduction in the concentration of chain ends in the amorphous region [35]. This phenomenon has a correlation with the increase in viscosity of the PBSeT samples after SSP. To determine the crystallinity of PBSeT, the heating enthalpies of the first and second T_m_ were calculated. The crystallinity went up as the heating enthalpy increased. This is because segments of the amorphous region were condensed through the SSP process, which was carried out at a temperature between T_g_ and T_m_, and this explains the increase in the crystallinity of PBSeT [42]. Compared with the control, all samples that underwent SSP had high crystallinity. Figure 7 shows the crystallinity of PBSeT samples after SSP at different times and temperatures. The degree of crystallinity in the sample that underwent SSP at 90 °C increased more rapidly over time than for other temperatures, resulting in the highest degree of crystallinity at 40 min. The samples that underwent SSP at 70 °C and 80 °C saw a gradual increase in crystallinity. The highest degree of crystallinity of PBSeT at 80 °C was higher than at 70 °C. This demonstrated that the ramifications of SSP were more favorable when the temperature of SSP increased. Unlike the heating enthalpy, the T_m_ value of PBSeT did not change significantly after the SSP process. These results may indicate that the increment in crystallinity is not related to the thermal properties. The onset decomposition temperature, T_onset_, and the maximum thermal degradation temperature, T_max_, were not improved after SSP and are located in a certain range of values, as shown in Table 1.

**Figure 8:**
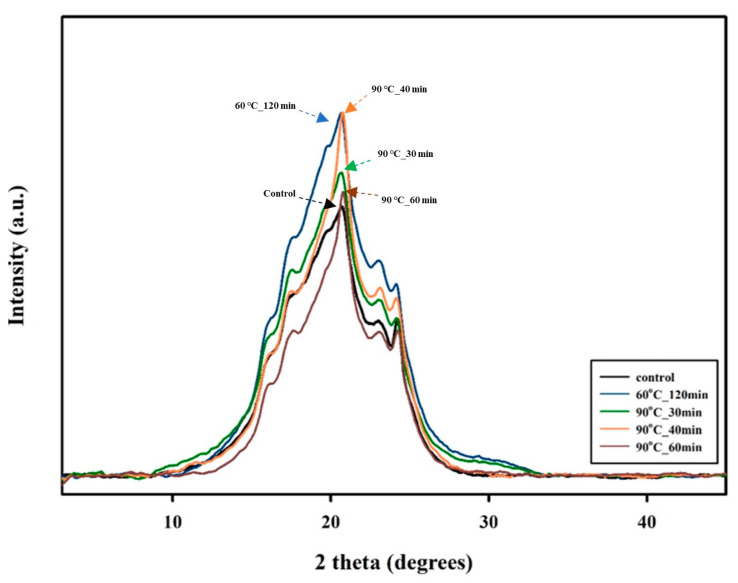
XRD data for PBSeT after SSP processing at 90 °C. Samples that underwent SSP processing at 60 °C for 120 min are added for comparison. The intensity of the peak increases as SSP time increases.

The crystallinity of PBSeT samples after SSP was also examined via XRD. Figure 8 shows that the synthesized PBSeT is more amorphous than other bio-based polyesters [42,43,44]; moreover, the increase in the intensity of the peak was verified. The diffraction signals of all the PBSeT samples are similar to those of synthesized PBSeT in a previous study [45]. The diffraction peaks at approximately 20° and 23° indicate crystal-type PBSe [20,22], and the diffraction peaks detected at 16°, 17°, and 24° are practically the same as that of the crystalline part of PBT [7]. The intensity of the XRD peaks tended to gradually rise during the first 40 min of SSP at 90 °C, and the crystallinity of PBSeT increased after the SSP process.

The sample that underwent SSP at 60 °C for 120 min showed a peak increment trend that was comparable to that of the sample at 90 °C for 40 min, although the treatment time was three times longer. This demonstrates that the temperature of SSP affects the SSP efficiency, which improves as the SSP temperature approaches the T_m_ of the polymer. The two-phase model for a semicrystalline polymer can explain the correlation between SSP and crystallinity. In the work of Kuran et al. [46], the two-phase model indicates that end groups of polymers in the amorphous phase have undergone a reaction during the SSP process. If the crystallinity of a polymer increases, the end groups in the amorphous phase become more concentrated and the SSP efficiency increases [47]. The peak intensity of the samples that underwent SSP for more than 40 min decreased slightly, and this trend shows a correlation with the *η* and the rheometer results. The chain segment in the crystalline phase of PBSeT may be degraded by heat energy over a specific duration of time [39].

The size of the crystallites was also investigated by XRD, and the results are given in Table 2. The size of the crystallites decreased from 11.8 to 10.7 Å during the first 40 min of SSP at 90 °C. The sample that underwent SSP at 60 °C for 120 min had smaller crystallites, but its required SSP duration was three times longer than that for the sample prepared at 90 °C. Therefore, the sample that underwent SSP at 90 °C showed better efficiency in improving the crystal structure. The reduction in crystalline size is correlated with the SSP rate. According to Wach et al. [48], the higher rate of SSP is accompanied by an increase in the number of polymer nucleation sites [49]. This phenomenon induced a faster polymerization of PBSeT; hence, the time may be insufficient to afford larger crystallites.

## 4. Conclusions

This study verified that SSP could be an effective method for improving the properties of PBSeT within a short period of time. PBSeT was successfully synthesized, and the effect of SSP on PBSeT at 70, 80, and 90 °C was confirmed through experiments. The results for viscosity and loss modulus showed changes in the rheological characteristics of PBSeT after SSP. From the results, SSP clearly improved the rheological properties of PBSeT. The values of the rheological properties such as *η* and complex viscosity increased with an increase in the SSP temperature. In addition, crystallinity data verified the improvement in the degree of crystallinity. According to the XRD analysis, it was confirmed that the crystallinity of PBSeT increased with the length of SSP processing time, and it can be concluded that SSP effectively improved the crystallinity of PBSeT. According to the DSC analysis, the PBSeT sample did not show a significant change in the T_m_ value with increasing SSP time and temperature of SSP. The increase in the crystallinity of PBSeT occurred independently of the thermal properties. The results of this paper indicate that SSP is an effective method for enhancing the properties of PBSeT and that SSP has the potential to modify the characteristics of PBSeT and other polyesters.

## Figures and Tables

**Table 1:** Thermal properties of PBSeT after SSP at different temperatures, obtained from DSC and TGA analysis.

Temperature (°C)	SSP Time (min)	T_g_ (°C)	T_m1_ ^a^ (°C)	T_m2_ ^b^ (°C)	H_m1_ ^c^ (J/g)	H_m2_ ^d^ (J/g)	X_c_ ^e^ (%)	T_onset_ (°C)	T_max_ (°C)
Control	0	−41.7	31.6	93.4	9.5	8.4	10.4	378.5	399.2
70	10	−41.8	31.1	91.1	9.1	9.2	10.8	377.1	405.2
	20	−42.5	31.3	95.3	9.6	9.0	10.9	375.6	401.9
	30	−42.9	31.5	93.9	9.4	9.2	10.9	378.3	400.5
	40	−43.5	31.2	93.6	9.7	9.1	11.0	380.1	399.8
	50	−41.2	32.8	98.5	10	9.4	11.4	376.4	403.9
80	10	−43.0	31.8	93.9	9.1	8.8	10.8	379.4	400.3
	20	−42.8	31.6	94.7	9.2	9.2	11.0	380.6	399.6
	30	−43.0	30.8	93.5	9.9	9.4	11.2	378.6	400.2
	40	−43.5	32.3	95.5	10.9	9.2	11.6	380.3	398.9
	50	−43.7	31.3	96.3	10.8	9.5	11.8	381.7	401.4
90	10	−43.9	31.5	95.0	8.9	9.7	11.0	375.1	403.4
	20	−42.5	31.5	93.9	9.9	9.5	11.3	378.2	401.0
	30	−43.5	31.4	96.2	9.9	9.7	11.5	382.1	403.7
	40	−44.0	31.2	96.3	10.7	9.9	12.0	381.0	404.3
	50	−43.9	31.3	95.9	10.5	10.0	11.9	379.7	400.8

^a^ First melting temperature of PBSeT. ^b^ Second melting temperature of PBSeT. ^c^ Enthalpy of fusion of first melting temperature peak. ^d^ Enthalpy of fusion of second melting temperature peak. ^e^ Degree of crystallinity of PBSeT.

**Table 2:** FWHM and crystallite size of PBSeT at main crystalline peak. The code for each sample is specified as SSP temperature_SSP processing time (e.g., 90_40 corresponds to the sample subjected to the SSP process at 90 °C for 40 min).

Sample	2 Theta (Degrees)	FWHM	D (Å)
Control	20.0	7.3	11.8
60_120	20.5	7.9	10.2
90_30	20.0	6.8	11.0
90_40	20.7	7.5	10.7
90_60	20.8	7.1	11.4

## Data Availability

Not applicable.

## References

[1] Esen G., AteÅŸ M. (2018). Architectural Vantage Point to Bioplastics in the Circular Economy. J. Archit. Res. Dev..

[2] Wang S., Daelemans L., D’Hooge D.R., Couck L., Broeck W.V.D., Cornillie P., Gou M., De Clerck K., Cardon L. (2021). Lifting the quality of fused filament fabrication of polylactic acid based composites. Compos. Part B Eng..

[3] Murariu M., Dubois P. (2016). PLA composites: From production to properties. Adv. Drug Deliv. Rev..

[4] Wang Y., Chen R., Cai J., Liu Z., Zheng Y., Wang H., Li Q., He N. (2013). Biosynthesis and Thermal Properties of PHBV Produced from Levulinic Acid by *Ralstonia eutropha*. PLoS ONE.

[5] Tan G.-Y.A., Chen C.-L., Li L., Ge L., Wang L., Razaad I.M.N., Li Y., Zhao L., Mo Y., Wang J.-Y. (2014). Start a Research on Biopolymer Polyhydroxyalkanoate (PHA): A Review. Polymers.

[6] Sharma V., Misra S., Srivastava A.K. (2017). Developing a green and sustainable process for enhanced PHB production by Azohydromonas australica. Biocatal. Agric. Biotechnol..

[7] Huang F., Wu L., Li B.-G. (2020). Sulfonated biodegradable PBAT copolyesters with improved gas barrier properties and excellent water dispersibility: From synthesis to structure-property. Polym. Degrad. Stab..

[8] Jian J., Xiangbin Z., Xianbo H. (2020). An overview on synthesis, properties and applications of poly (butylene-adipate-co-terephthalate)–PBAT. Adv. Ind. Eng. Polym. Res..

[9] Cai Y., Lv J., Feng J. (2012). Spectral Characterization of Four Kinds of Biodegradable Plastics: Poly (Lactic Acid), Poly (Butylenes Adipate-Co-Terephthalate), Poly (Hydroxybutyrate-Co-Hydroxyvalerate) and Poly (Butylenes Succinate) with FTIR and Raman Spectroscopy. J. Polym. Environ..

[10] Liu L., Yu J., Cheng L., Yang X. (2009). Biodegradability of poly(butylene succinate) (PBS) composite reinforced with jute fibre. Polym. Degrad. Stab..

[11] Herrera R., Franco L., Rodríguez-Galán A., Puiggalí J. (2002). Characterization and degradation behavior of poly(butylene adipate-co-terephthalate)s. J. Polym. Sci. Part A Polym. Chem..

[12] Li G., Shankar S., Rhim J.-W., Oh B.-Y. (2015). Effects of preparation method on properties of poly(butylene adipate-co-terephthalate) films. Food Sci. Biotechnol..

[13] Castellan A., Bart J.C.J., Cavallaro S. (1991). ChemInform Abstract: Industrial Production and Use of Adipic Acid. Cheminform.

[14] Qin P., Wu L., Li B., Li N., Pan X., Dai J. (2021). Superior Gas Barrier Properties of Biodegradable PBST vs. PBAT Copolyesters: A Comparative Study. Polymers.

[15] Heidarzadeh N., Rafizadeh M., Taromi F.A., del Valle L.J., Franco L., Puiggalí J. (2017). Biodegradability and biocompatibility of copoly(butylene sebacate-co-terephthalate)s. Polym. Degrad. Stab..

[16] Liu T., Li Z., Jiang T., Xi S., Li Y., Guo J., Huang M., Algadi H., Ye X., Jiang Q. (2022). Improvement of thermodynamic properties of poly(butanediol sebacate-butanediol terephthalate) (PBSeT) composites based on the dispersion of PCaCO3@tannic acid formed by complexation of tannic acid and Ti. Adv. Compos. Hybrid Mater..

[17] Nouruzi N., Dinari M., Gholipour B., Mokhtari N., Farajzadeh M., Rostamnia S., Shokouhimehr M. (2021). Photocatalytic hydrogen generation using colloidal covalent organic polymers decorated bimetallic Au-Pd nanoalloy (COPs/Pd-Au). Mol. Catal..

[18] Jayaramulu K., Narayanan R.P., George S.J., Maji T.K. (2012). Luminescent Microporous Metal–Organic Framework with Functional Lewis Basic Sites on the Pore Surface: Specific Sensing and Removal of Metal Ions. Inorg. Chem..

[19] Eshghi F., Mehrabadi Z., Farsadrooh M., Hayati P., Javadian H., Karimi M., Karimi-Maleh H., Rostamnia S., Karaman C., Aghababaei F. (2023). Photocatalytic degradation of remdesivir nucleotide pro-drug using [Cu (1-methylimidazole) 4 (SCN) 2] nanocomplex synthesized by sonochemical process: Theoretical, hirshfeld surface analysis, degradation kinetic, and thermodynamic studies. Environ. Res..

[20] Jaisankar V., Nanthini R., Karunanidhi M., Ravi A. (2010). Study on biodegradable random copolyesters derived from 1, 4-butane diol, terephthalic acid and adipic acid/sebacic acid. Asian J. Chem..

[21] Kim S.J., Kwak H.W., Kwon S., Jang H., Park S.-I. (2020). Synthesis, Characterization and Properties of Biodegradable Poly(Butylene Sebacate-*Co*-terephthalate). Polymers.

[22] Li Z., Li Y., Dong X., Wang W., Zhu Y.-C., Murugadoss V., Song G., Naik N., Pan D., Guo Z. (2021). Synthesis, characterization and properties of poly(butanediol sebacate–butanediol terephthalate) (PBSeT) copolyesters using glycerol as cross-linking agent. Mater. Today Commun..

[23] Kwon S., Kim Y., Jang H., Kim S.J., Park S.I. (2023). Poly (ethylene oxide)(PEO) influence on mechanical, thermal, and degradation properties of PLA/PBSeT blends. J. Appl. Polym. Sci..

[24] Jang H., Kwon S., Kim S.J., Park S.-I. (2022). Maleic Anhydride-Grafted PLA Preparation and Characteristics of Compatibilized PLA/PBSeT Blend Films. Int. J. Mol. Sci..

[25] Erber M., Boye S., Hartmann T., Voit B.I., Lederer A. (2009). A convenient room temperature polycondensation toward hyperbranched AB_2_ -type all-aromatic polyesters with phenol terminal groups. J. Polym. Sci. Part A Polym. Chem..

[26] Vouyiouka S., Theodoulou P., Symeonidou A., Papaspyrides C.D., Pfaendner R. (2013). Solid state polymerization of poly(lactic acid): Some fundamental parameters. Polym. Degrad. Stab..

[27] Li H., Shang Y., Huang W., Xue B., Zhang X., Cui Z., Fu P., Pang X., Zhao Q., Liu M. (2021). Synthesis of succinic acid-based polyamide through direct solid-state polymerization method: Avoiding cyclization of succinic acid. J. Appl. Polym. Sci..

[28] Cruz S.A., Zanin M. (2005). PET recycling: Evaluation of the solid state polymerization process. J. Appl. Polym. Sci..

[29] Medellin-Rodriguez F.J., Lopez-Guillen R., Waldo-Mendoza M.A. (2000). Solid-state polymerization and bulk crystallization behavior of poly (ethylene terephthalate)(PET). J. Appl. Polym. Sci..

[30] Agrawal A.K., Mhaisgawali V.T. (2006). Post-extrusion solid-state polymerization of fully drawn polyester yarns. J. Appl. Polym. Sci..

[31] Chronaki K., Korres D.M., Papaspyrides C.D., Vouyiouka S. (2020). Poly(lactic acid) microcapsules: Tailoring properties via solid state polymerization. Polym. Degrad. Stab..

[32] Beltrán F.R., Climent-Pascual E., de la Orden M.U., Urreaga J.M. (2020). Effect of solid-state polymerization on the structure and properties of mechanically recycled poly(lactic acid). Polym. Degrad. Stab..

[33] Uvarov V., Popov I. (2007). Metrological characterization of X-ray diffraction methods for determination of crystallite size in nano-scale materials. Mater. Charact..

[34] Kim S.J., Kwak H.W., Kwon S., Jang H., Park S.-I. (2021). Characterization of PLA/PBSeT Blends Prepared with Various Hexamethylene Diisocyanate Contents. Materials.

[35] Papadopoulos L., Xanthopoulou E., Nikolaidis G., Zamboulis A., Achilias D., Papageorgiou G., Bikiaris D. (2020). Towards High Molecular Weight Furan-Based Polyesters: Solid State Polymerization Study of Bio-Based Poly(Propylene Furanoate) and Poly(Butylene Furanoate). Materials.

[36] Panagiotopoulos C., Porfyris A., Korres D., Vouyiouka S. (2020). Solid-State Polymerization as a Vitrimerization Tool Starting from Available Thermoplastics: The Effect of Reaction Temperature. Materials.

[37] Xi Z., Liu T., Si W., Bi F., Xu Z., Zhao L. (2018). High-efficiency acetaldehyde removal during solid-state polycondensation of poly(ethylene terephthalate) assisted by supercritical carbon dioxide. Chin. J. Chem. Eng..

[38] Kim J., Roberts G.W., Kiserow D.J. (2008). Effect of prepolymer molecular weight on solid state polymerization of poly(bisphenol a carbonate) with nitrogen as a sweep fluid. J. Polym. Sci. Part A Polym. Chem..

[39] Gross S.M., Roberts G.W., Kiserow D.J., DeSimone J.M. (2001). Synthesis of High Molecular Weight Polycarbonate by Solid-State Polymerization. Macromolecules.

[40] Dawin T.P., Ahmadi Z., Taromi F.A. (2019). Biocompatible PLA/PHB coatings obtained from controlled solid state polymerization. Prog. Org. Coatings.

[41] Sadiku-Agboola O., Sadiku E.R., Adegbola A.T., Biotidara O.F. (2011). Rheological Properties of Polymers: Structure and Morphology of Molten Polymer Blends. Mater. Sci. Appl..

[42] Devotta I., Mashelkar R. (1993). Modelling of polyethylene terephthalate reactors—X. A comprehensive model for solid-state polycondensation process. Chem. Eng. Sci..

[43] Li L.-J., Duan R.-T., Zhang J.-B., Wang X.-L., Chen L., Wang Y.-Z. (2013). Phosphorus-Containing Poly(ethylene terephthalate): Solid-State Polymerization and Its Sequential Distribution. Ind. Eng. Chem. Res..

[44] van der Wal A., Mulder J., Gaymans R. (1998). Fracture of polypropylene: The effect of crystallinity. Polymer.

[45] Feng Y., Li Y., Ye X., Li Z., Wang W., Liu T., El Azab I.H., Mersal G.A.M., Ibrahim M.M., El-Bahy Z.M. (2022). Synthesis and characterization of 2,5-furandicarboxylic acid poly(butanediol sebacate-butanediol) terephthalate (PBSeT) segment copolyesters with excellent water vapor barrier and good mechanical properties. J. Mater. Sci..

[46] Kuran W., Dębek C., Wielgosz Z., Kuczyńska L., Sobczak M. (2000). Application of a solid-state postpolycondensation method for synthesis of high molecular weight polycarbonates. J. Appl. Polym. Sci..

[47] Zimmerman J. (1964). Equilibria in solid phase polyamidation. J. Polym. Sci. Part B Polym. Lett..

[48] Wach R.A., Wolszczak P., Adamus-Wlodarczyk A. (2018). Enhancement of Mechanical Properties of FDM-PLA Parts via Thermal Annealing. Macromol. Mater. Eng..

[49] Chen J., Deng C., Hong R., Fu Q., Zhang J. (2020). Effect of thermal annealing on crystal structure and properties of PLLA/PCL blend. J. Polym. Res..

